# Multifaceted Activity of Fabimycin: Insights from Molecular Dynamics Studies on Bacterial Membrane Models

**DOI:** 10.1021/acs.jcim.4c00228

**Published:** 2024-05-11

**Authors:** Mateusz Rzycki, Dominik Drabik

**Affiliations:** Department of Biomedical Engineering, Wroclaw University of Science and Technology, Wroclaw 50-370, Poland

## Abstract

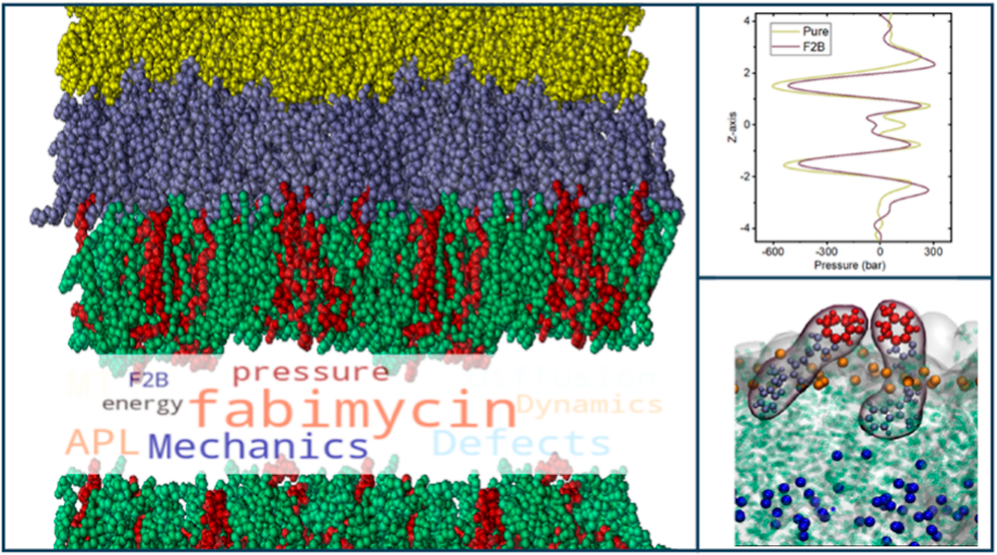

Membranes—cells’ essential scaffolds—are valid molecular targets for substances with an antimicrobial effect. While certain substances, such as octenidine, have been developed to target membranes for antimicrobial purposes, the recently reported molecule, fabimycin (F2B)—a novel agent targeting drug-resistant Gram-negative bacteria—has not received adequate attention regarding its activity on membranes in the literature. The following study aims to investigate the effects of F2B on different bacterial membrane models, including simple planar bilayers and more complex bilayer systems that mimic the *Escherichia coli* shell equipped with double inner and outer bilayers. Our results show that F2B exhibited more pronounced interactions with bacterial membrane systems compared to the control PC system. Furthermore, we observed significant changes in local membrane property homeostasis in both the inner and outer membrane models, specifically in the case of lateral diffusion, membrane thickness, and membrane resilience (compressibility, tilt). Finally, our results showed that the effect of F2B differed in a complex system and a single membrane system. Our study provides new insights into the multifaceted activity of F2B, demonstrating its potential to disrupt bacterial membrane homeostasis, indicating that its activity extends the currently known mechanism of FabI enzyme inhibition. This disruption, coupled with the ability of F2B to penetrate the outer membrane layers, sheds new light on the behavior of this antimicrobial molecule. This highlights the importance of the interaction with the membrane, crucial in combating bacterial infections, particularly those caused by drug-resistant strains.

## Introduction

Cell membranes, composed of lipid bilayers, play a pivotal role in maintaining the integrity and functionality of living cells.^1^ The dynamic and complex nature of these membranes presents a major challenge for theoretical studies, particularly in the context of molecular dynamics (MD) simulations.^2,3^ Although MD simulations have proven invaluable in studying the behavior of lipid membranes, they often struggle to accurately reflect the intricacies of real membrane systems, particularly those in bacteria.^4,5^ Thus, simulations with more complex systems that can better mimic biological membranes are needed.

Despite the success of MD simulations in modeling simple lipid bilayers, such systems tend to neglect critical aspects of real biological membranes. A workaround for this problem is the use of heterogeneous membrane systems.^6,7^ Nevertheless, the use of small, symmetric lipid bilayers may not adequately capture the heterogeneous lipid compositions and asymmetric arrangements observed in bacterial membranes.^8^ Furthermore, these simplified models may not fully represent the dynamic nature of lipid–protein interactions or the influence of membrane curvature and lateral organization on cellular processes.^9^ As a result, there is an urgent need to develop simulations with more realistic and complex membrane systems to achieve a deeper understanding of cellular processes at the molecular level.^4,10^ In recent years, researchers have made commendable efforts to address these limitations and model the behavior of a crowded environment of bacteria membrane systems using an in silico approach.^11−13^ Some of them shed light on how antimicrobials act on bacterial components, providing valuable information for developing new antibiotics that target bacterial infections.^11^ A notable and promising advancement in antimicrobial research is fabimycin (F2B)—a groundbreaking agent tailored to combat drug-resistant bacteria.^14^ It specifically targets Gram-negative pathogens known for their robust outer membranes that hinder the penetration of drugs. It belongs to a class of antimicrobials that has been confirmed to target the FabI enzyme, a critical component in the biosynthesis of bacterial fatty acids. F2B is a Debio-1452 derivative and offers promise to counter the increasing threat of drug-resistant Gram-negative bacteria in healthcare.^14^

In our work, we investigate the multifaceted activity of F2B beyond its conventional targeting of FabI enzymes. To accomplish this, we performed an extensive series of molecular dynamics studies on various bacterial membrane models, including planar and complex bilayers consisting of both outer and inner membranes. Our study investigated the selectivity and behavior of F2B in these different membrane environments, complemented by energetic analysis using the umbrella sampling method. Our findings demonstrate that F2B not only exhibits a higher affinity for bacterial membranes but also induces local perturbations of key membrane properties. Moreover, it exerts a significant influence on the membrane pressure, especially in complex systems. Most importantly, our results provide compelling evidence that the activity of F2B extends beyond the conventional targeting of the FabI enzyme, highlighting its multifaceted potential as a versatile agent to combat bacterial infections.

## Methods and Simulation Details

### Fabimycin Parametrization

The initial structure of F2B (see Figure S1) was received using SCIGRESS software (SCIGRESS, Molecular modeling software, FQS Poland, ver. FJ-3.3.3), in which the primary geometry, hybridization, and net charge were optimized. During the modeling process, we followed the representation of the molecule reported by Parker et al.^14^ Consequently, F2B exhibits a cationic character with a net charge of +1 (positively charged amine group). The initial configuration was further evaluated in the CHARMM General Force Field^15^ to deliver the best possible template for further optimization. Quantum level calculations were performed by using the Gaussian 2016 software package. The equilibrium geometry of F2B was calculated at the MP2/6-31G(d) level of theory. Partial atomic charges were optimized on the basis of calculated water-interaction sites consistent with the existing CHARMM force field parameters.^16,17^ The distance between water molecules and target atoms and the rotation angle of water molecules were optimized quantum mechanically at the HF/6-31G(d) level of theory and further fitted using the Force Field Toolkit.^17^ Supplementary analysis based on the construction of the quantum mechanical (QM) Hessian matrix (the matrix of second derivatives of the energy with respect to geometry) was also performed at the B3LYP level of theory for further use in the force field parametrization for further molecular dynamics (MD) study.^18,19^ Specific geometric data such as bond lengths, angles, and dihedrals were extracted from a QM potential energy surface (PES). These parameters are crucial for the construction of the force field used in MD simulations. The conversion of molecular topology (from CHARMM to GROMACS) was performed with TopoGromacs.^20^

### Molecular Dynamics Simulations

Membrane models were constructed using the Membrane Builder tool in CHARMM-GUI.^21−24^ The exact composition of the models and selected lipid structures is presented in Table S1 and Figure S1, respectively. To mimic the ionic environment in bacteria, a 0.22 M sodium chloride (NaCl) solution with the TIP3P water model was employed. GROMACS (v. 2021) with the CHARMM36 force field was used for all-atom simulations^25,26^ following standard six-step CHARMM-GUI process. Initially, the systems passed the energy minimization and equilibration process. The equilibration procedure involved a constant number of particles, volume, and temperature (*NVT*) ensemble with a time step of 1 fs for 250 ps. The temperature was controlled using the Nose–Hoover thermostat at 303.15 K with a time constant (τ_t_) of 1 ps.^27,28^ That was followed by *NPT* dynamics with a 1 fs integration for 250 ps and with a 2 fs integration step for 5 ns. The pressure was maintained at 1 bar using a semi-isotropic coupling via the Parinello–Rahman barostat with a time constant (τ_p_) of 5 ps.^29^ During equilibration, positional, and dihedral restraints were gradually reduced. van der Waals interactions were truncated at 1.2 nm with a force-based switching function,^30^ while long-range electrostatics were computed using the particle mesh Ewald (PME) method.^31^ Each membrane simulation was repeated three times with random initial velocities to ensure reproducibility of the obtained results. The average simulation time for each replica is shown in Table S1. The stability of the APL values was considered as an indication of the equilibration process. In Figure S2, the maintenance of the surface area per lipid in the simulation time fragment is presented.

The potential of the mean force (PMF) of F2B molecules along the reaction coordinate was calculated using the umbrella sampling method. The reaction coordinate was defined as the distance between the center of mass (COM) of the antimicrobial agent and the center of mass of the membrane projected along the bilayer normal (*z* direction). Initially, steered molecular dynamics simulations were conducted to obtain the starting configurations for umbrella sampling. During this phase, the antimicrobial agent was pulled from the bulk water toward the center of the bilayer. To ensure sufficient sampling, a minimum of 80,000 samples were collected for each step, with a spacing of 0.1 nm between subsequent windows. In some cases, additional simulations were performed to enhance the sampling. Subsequently, a brief (5 ns) equilibration was performed using both the *NVT* and *NPT* ensembles for each window. The actual umbrella sampling simulations were then conducted with a force constant of 1000 kJ mol^–1^ nm^–2^, applying a harmonic potential to restrain the agent along the reaction coordinate. Each window was simulated for at least 50 ns. The weighted histogram analysis method (WHAM) (see Figure S3), integrated into the GROMACS software, was utilized to determine the free energy profile.^32^

### In Silico Membrane System Characteristics

#### Area per Lipid and Membrane Thickness

Both the area per lipid (APL) and the membrane thickness (MT) were determined by using a custom Matlab script. The midpoint position between selected head atoms was used as a point position for each lipid. This was followed by Voronoi tessellation to obtain individual APL for each lipid molecule in each simulation time step. The overall membrane APL is determined by Gaussian fitting of the histogrammed APL values from all molecules and is obtained by averaging for a specific molecule type. The MT was computed as the difference between the average height (*z*-axis) values of the atoms in opposing leaflets. The midpoint for both APL and MT determination was calculated from position of P and C2 atoms (notably P and C2 for PYPE and PYPG, P1 and P3 for PVCL, and PA and PB for LipidA).

#### Bending Rigidity and Tilt Rigidity

Mechanical properties, including the bending rigidity coefficient and tilt modulus, were assessed using a real-space fluctuation (RSF) method.^33^ This involved determining the probability distribution of tilt and splay for all lipids across all analyzed timesteps. Tilt θ is defined as an angle between the lipid director [vector between lipid head, midpoint between head atoms (defined in APL section), and lipid tail, midpoint between last carbon atoms] and bilayer normal. Lipid splay is defined as divergence of an angle formed by the directors of neighboring lipids providing that they are weakly correlated. Lipid tail atoms are defined as follows: C216 and C316 for PYPE and PYPG; CA14, CB14, CC14, and CD14 for PVCL; C66, C55, C710, C712, and C114 for LipidA.

#### Compressibility

Following the methodology of Doktorova et al.,^34,35^ the compressibility modulus of lipid membranes was determined. Briefly, fluctuations of membrane thickness are used as a mean to determine leaflet compressibility modulus *K*_A_.

#### Lateral Diffusion Coefficient

The internal function *gmx msd* in Gromacs was employed to calculate the 2D lateral diffusion of the lipid molecules. This calculation is based on Einstein’s relation, utilizing the mean square displacement (MSD) of selected molecular species (P and N14 atoms in this study) in 50 ns blocks.^36^ While it was reported that finite size effects and hydrodynamics might contribute to difference between experimentally and computationally determined diffusion, systems constructed in this study (with exception for LPS leaflet of outer membrane) are sufficiently large to maintain good agreement with experiments.^37,38^

#### Interdigitation

Interdigitation parameter was determined using MEMBPLUGIN.^39^ The obtained parameter is defined as the width of the region of mass overlap.

#### Order Parameter

The carbon–hydrogen (deuterium) order parameter, denoted as |*S*_cd_|, is defined by eq 1
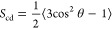
1Here, θ represents the angle between the carbon–hydrogen vector on a lipid molecule and the normal to the bilayer (*z*-direction). The angular brackets ⟨⟩ indicate that the value is a time-averaged measure.

#### Acyl Chain Accessibility

Adopting the protocol by Boyd et al.^40^ acyl chain accessibility was determined. Briefly, this involved calculating the occupancy of each bilayer atom based on van der Waals radius, labeling headgroup atoms as polar and others as tail. Headgroup is defined as all atoms under the C2 atom or equivalent. This is followed by dividing the simulation box into square grids with a spacing of 0.5 Å. The occupancy graph at each grid position indicates whether it is part of the head or tail region, aiding in identifying the bilayer surface fraction that allows acyl chain access. Sphere probe analysis, ranging from 1 to 5 nm in radius, is then conducted to discount minor gaps in headgroup coverage, ensuring that only significant hydrophobic patches are classified as true defects.

#### Membrane Curvature

Membrane curvature was computed using the MDAnalysis^41,42^ package, a Python library widely utilized in molecular dynamics simulations. We employed the MembraneCurvature module within MDAnalysis,^43^ which calculates the mean curvature based on a selection of reference atoms. In this case, the surface of the reference was determined by the *z* position of phosphorus atoms within each leaflet of the membrane.

#### Lateral Pressure Profiles

The study employed GROMACS-LS,^44^ a customized version of GROMACS, to calculate the local stress tensor utilizing the central force decomposition (CFD) method and to derive the lateral and normal stress profiles of the bilayer. The last 100 ns of trajectories were adapted to comply with the software requirements; thus, the calculations of the PME electrostatic forces were settled to cutoff. The long-range electrostatic contribution to local stress has been considered only up to a cutoff radius of 2.6 nm based on recent insights.^45^ The lateral component of the pressure tensor, denoted as *P*_L_(*z*), was calculated using the expression . Similarly, the normal component of the pressure tensor, represented as *P*_N_(*z*), was determined as *P*_N_(*z*) = *P*_*zz*_(*z*). Finally, LPP was computed by subtracting the normal component from the lateral component over the *Z*-axis (eq 2).
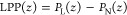
2To discuss further the behavior of membrane systems, the area under the LPP was determined according to eq 3.
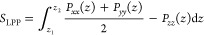
3where *z*_1_ and *z*_2_ denote the upper and lower boundaries of the simulation box on the *Z* axis.

The analyses were performed on the last 100 ns of each system replica.

### Statistics

In this study, we primarily evaluated the impact of F2B on various properties by comparing the differences between systems before and after its incorporation. This was achieved by subtracting the value of the system with F2B from the corresponding system without. To determine the statistical significance of these differences, we predominantly employed the one-way ANOVA test, setting the significance threshold at 0.05, unless otherwise specified. For the post hoc analysis, the Tukey test was utilized. Statistical analyses were performed using OriginPro 2015 software from OriginLabs. The results are presented as mean values derived from three system replicas along with their respective standard deviations, while the PMF and pressure profile errors were assessed by using the bootstrap method.

## Results

### Inner Membrane

**Figure 1:**
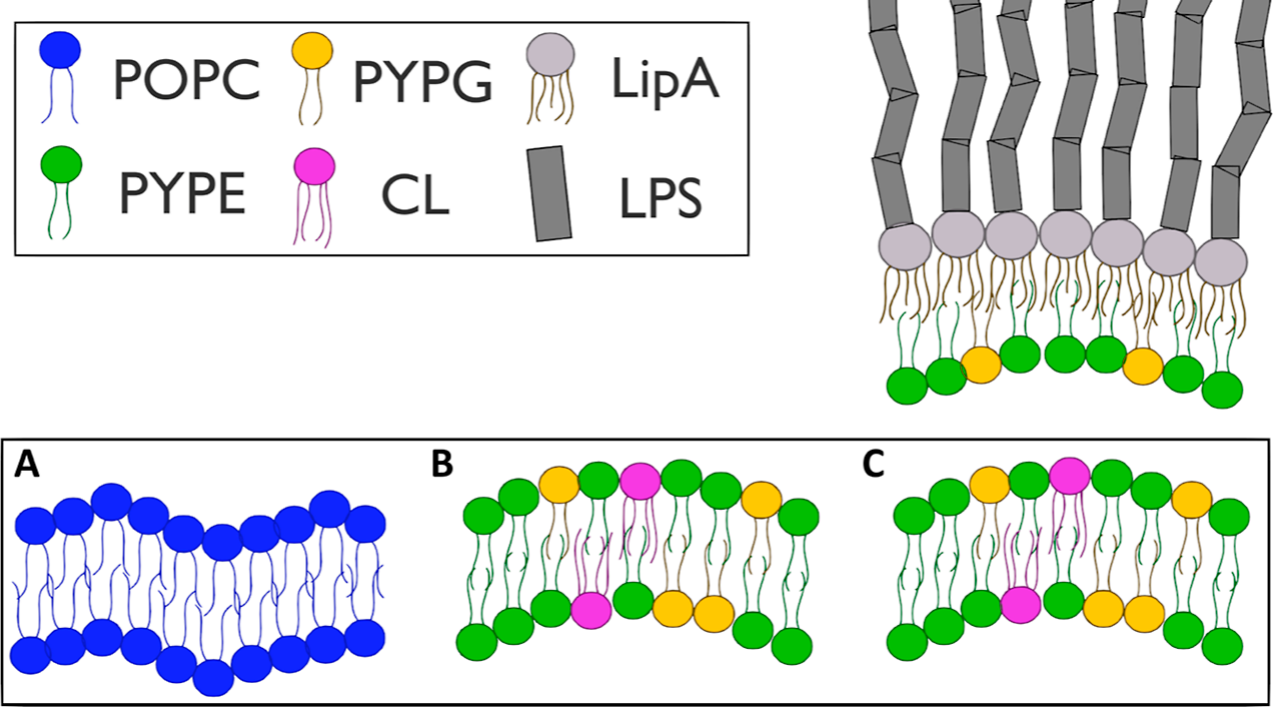
Sketch visualizing expansion of inner membrane model from (A) PC control, (B) single IM (sIM) membrane, and (C) inner membrane in a complex (cIM) two-membrane system. Lipids are colored according to the type, POPC—blue, PYPE—green, PYPG—yellow, cardiolipin (CL)—violet, LipidA—gray, and LPS—dark gray. The black square represents membranes from the system analyzed in this subchapter.

To thoroughly assess the impact of F2B on biological membranes and to discern variations in membrane dynamics based on complexity, we conducted a detailed analysis of membrane properties in the presence and absence of F2B. This analysis differentiates between the inner and outer membranes due to their distinct roles in bacteria. The *Escherichia coli* inner membrane, a widely utilized model for simulations studying particle effects on bacterial membranes. This model has been extensively used in our previous research and is well-recognized in the scientific community, as evidenced by several publications in the field.^46−49^ In Figure 1, we present schematic illustrations of the evaluated systems, including the homogeneous phosphatidylcholine (PC) system as a neutral control, a single inner membrane (sIM) system representing a classical approach, and the inner membrane of a complex system (cIM). Our complex system comprises both inner and outer membranes separated by a water slab mimicking periplasm. The lipid compositions of cIM and sIM remain the same.

**Figure 2:**
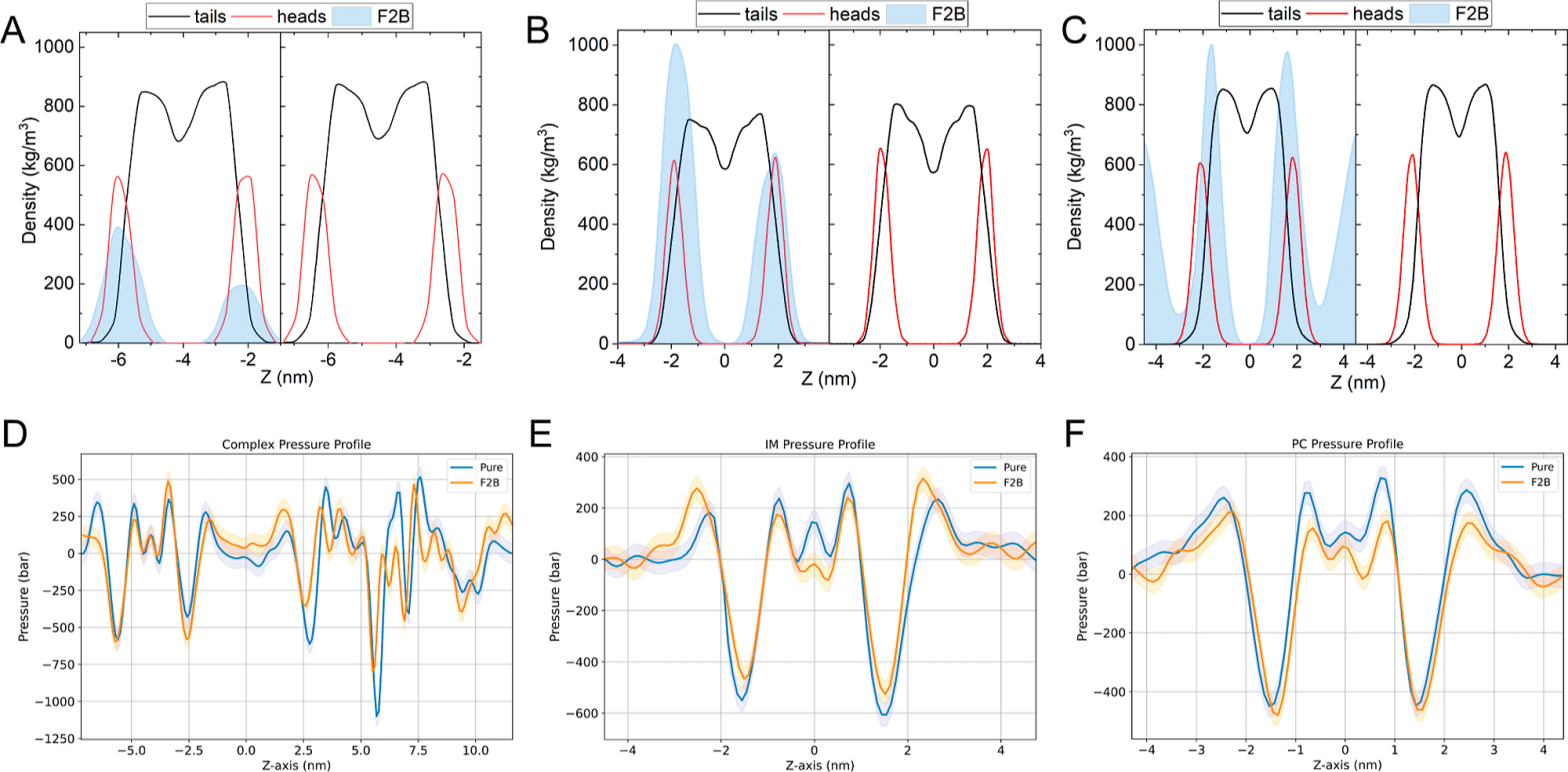
Comparison of mass density and lateral pressure profiles in various membrane systems. (A,D) Inner membrane of complex system (cIM), (B,E) single inner membrane (sIM), and (C,F) control POPC membrane (PC). The upper panels illustrate the spatial distribution of molecules within each system (with and without F2B on the left and right, respectively). The F2B mass density was normalized in each system for clarity. The lower panels depict the corresponding pressure profiles, highlighting the variations in lateral pressure across the pure and F2B-affected membranes.

Before examining the effects of F2B, we validated the integrity of the systems. Initially, we analyzed the mass density and pressure profiles of the investigated systems. The density of all investigated systems followed typical distribution characteristics of membrane systems. A preliminary examination of density plots revealed a notable phenomenon (Figure 2). The acyl chain region’s density in a sIM system is lower than that in the corresponding cIM system, while the head region density is higher. This trend was also observed in systems with F2B (Figure 2B).

One possible explanation for this difference is that in the complex system, where two membranes are simultaneously present, the outer membrane area and rigidity are higher; therefore, it dictates the box size, leading to a dilution effect (higher APL to cover whole area) in the inner membrane’s lipid head region. On the other hand, the systems were designed with matched lipid area (based on PBC) to counter this effect. Therefore, together with higher density of acyl chains, this indicates some effect that is not observable in a standard single system. At this stage, differences are already evident in the interactions of F2B with PC and sIM, marked by the agent’s distribution density. The location and orientation of F2Bs is further shown in Figures S4 and S5. In the PC system, F2B molecules are highly dispersed between the water and lipid layers, while in the IM system, they associate closely with the membrane, binding to the head and hydrophobic tail regions. Given the importance of bilayer pressure balance in such studies,^50,51^ we decided to further investigate this aspect through calculated lateral pressure profiles. In Figure 2C–F, pressure profiles are presented for the control, sIM, and cIM systems, respectively. Furthermore, to gain a comprehensive understanding of the behavior of the entire membrane system, we performed an integration over the LPP. This involved calculating the absolute area under the curve (AUC) to quantify the total impact of pressure changes across the membrane (Table 1).

**Table 1:** Absolute Area under LPP for sIM, cIM, and PC before and after F2B Exposure

		difference in PURE and F2B per leaflet [bar·nm]		
system	difference between leaflets [bar·nm]	inner leaflet	outer leaflet	pressure difference [bar·nm]
PC				
PURE	32.78 ± 9.19	95.09 ± 7.14	262.79 ± 19.73	357.88 ± 26.87
F2B	134.92 ± 9.83			
sIM				
PURE	69.99 ± 7.74	274.64 ± 11.62	244.33 ± 10.33	518.97 ± 21.95
F2B	39.68 ± 7.83			
cIM				
PURE	2.49 ± 9.61	239.92 ± 15.68	157.57 ± 10.30	397.49 ± 25.98
F2B	84.84 ± 8.73			

In cIM, sIM, and PC membranes, we can observe similar constituent trends over the whole profile with characteristic refractions from appropriate membrane regions. Our results follow those reported previously^44,52,53^ with some fluctuations resulting from, i.e., differences in force fields or the size of the simulated systems. The initial peak at the interface of the aqueous and lipid phases reflects the repulsive forces from membrane heads, while the wells denote attractive forces, indicating a tendency to minimize the membrane area. A further peak close to the bilayer center is characteristic of unsaturated lipid tails and reveals their double bond level. The pure cIM and sIM systems, despite their strong negative charge, maintain the pressure balance between the leaflets. The introduction of F2B into the PC system led to changes in the pressure profile at both the head and tail levels. The absolute pressure difference throughout the profile after F2B treatment was ∼358 bar·nm. All positive peaks were suppressed, resulting in a slightly reduced MT. Despite only limited binding of F2B to the membrane, the pressure in the hydrophobic tail region was also affected. In the IM system, a slightly different effect of F2B is visible. There is a translocation of pressure in the area of hydrophilic heads, which is accompanied by an increase in repulsive forces and a reduction of attractive ones. F2B activity resulted in a difference of ∼519 bar·nm, hence compared to PC. Taking into account the density profiles and tight agent binding at the level of lipid heads and tails, it can be concluded that the compound indicates a strong affinity for bacterial membranes and affects their pressure balance. In the context of cIM, translocation is observable and is accompanied by the formation of a high-pressure region on the inner leaflet at the level of unsaturated chains. In particular, the outer leaflet, initially enriched with F2B, exhibited a loss of its characteristic positive peak originating from the lipid heads. This observation additionally suggests a potential attraction of cationic F2B toward negatively charged membranes.

The observed disparities may originate from the inherent properties of F2B as a molecule or alternatively may result from the uneven incorporation of the compound into the membrane. It is credible that both factors contribute, as evidenced by density profiles that indicate an uneven distribution of F2B between lipid bilayer leaflets in both single and complex systems. We suppose that F2B redistribution could potentially alter membrane pressure/thermodynamic equilibrium, representing a plausible mechanism by which F2B may not only interfere with fatty acid production^14^ but also ultimately form pores. In contrast, the situation was notably distinct in the control PC system, where F2B was uniformly distributed across both leaflets. However, additional peaks appeared outside of the membrane system in the water phase. This observation highlights an enhanced affinity of F2B for the IM systems compared to the control since no such grouping outside the membrane was observed.

After examining the pressure profiles, another interesting dependency can be found. The lateral pressure is slightly higher in the sIM system than that in the cIM system. It was shown that when the lateral pressure increases, the polar headgroups come closer together and as a result, the access of hydrophobic parts decreases.^54^ This provides another interesting explanation for the decrease in the density in the headgroup region. Thus, it can be concluded that F2B maintains a specific mode of action, inducing a pressure disturbance concerning the reference membrane.

To verify our systems, we compared mainstream membrane properties to data available in the literature. We observed that the measured membrane thickness (MT, for POPC 3.92 ± 0.01 nm) and area per lipid (APL, for POPC 63.59 ± 0.22 Å^2^) of POPC fall between values reported previously in the literature.^35,55^ The comparison of IM systems is slightly more challenging due to the diverse composition of membranes mimicking bacterial ones. Nonetheless, those primarily composed of PE, PG, and CL maintain a similar trend in terms of MT (3.96 ± 0.03 nm), APL (58.1 ± 0.31 Å^2^), and acyl chain order (*S*_cd_).^46,47,56^ Furthermore, we conducted a thorough comparison between the cIM and sIM systems (see Figures S6–S10), finding no significant differences in the main membrane properties such as MT, APL, or *S*_cd_, which also remained consistent with the literature reports.

After a thorough characterization of the systems studied, an in-depth analysis of F2B’s activity was carried out. Antimicrobial molecules, such as octenidine, was shown to vary significantly in their aggregation behavior or molecular shape depending on the type of membrane.^46^ In the sIM system, all F2B particles incorporated into the bilayer, indicating a higher affinity to the lipid phase rather than the water environment. Most of the F2B molecules anchored to the membrane in the lance shape, directing benzofuran moieties toward the lipid acyl chain region. The other side of the F2B chain equipped with charged amine substituents targeted the water phase, lying at the headgroup level or slightly above. This arrangement may result from electrostatic interactions that direct the cationic amine group toward the negatively charged phosphate group. In general, F2B anchored to the membrane with an angle ranging from 40 to 60° with respect to bilayer normal. The distributions of positions adopted by F2B at incorporation into the membrane and the snapshots of such orientations are shown in Figure S5. Analogous behavior of F2B was also observed in the cIM system. The most notable differences were found when F2B interacting with the neutral POPC membrane. Although the shape and orientation of the incorporated F2B remained unchanged, the number of incorporated molecules was lower (see Figure S4). Only 60% of the total number of F2B molecules anchored to the membrane, while the rest remained in the water phase, forming aggregates or temporarily approaching the bilayer surface. These clusters lasted for about 50 ns, and tended to disintegrate afterward. This indicates a higher affinity of F2B for the negatively charged lipid phase rather than for the neutral one. In addition, we verified whether the F2B activity affects membrane curvature. For the sIM, cIM, and PC systems, we found no significant differences when modulating any curvature (see Figure S11).

**Figure 3:**
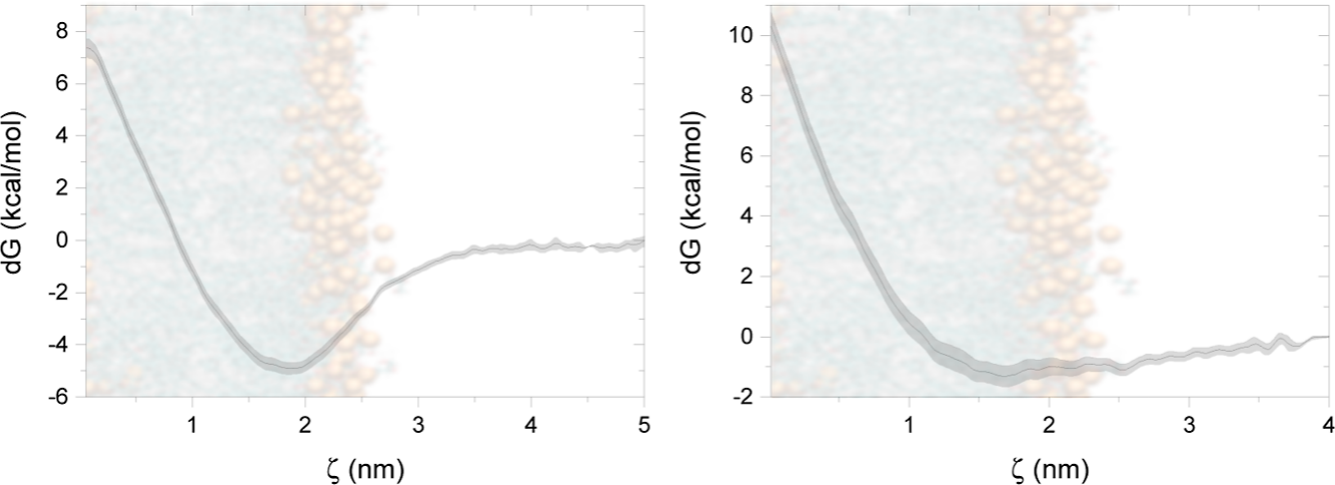
PMF profiles for F2B as a function of the distance from the center of IM (left) and PC (right) bilayer.

Both the pressure profiles and the behavior of the molecules indicated a stronger affinity of F2B for IM membrane systems. Nevertheless, a US analysis was performed to see the localization of Gibbs energy minima for each of the investigated systems. The free energy plots can be recognized as a general type according to the classification proposed by Neale and Pomes based on the interactions of small molecules with membranes.^57^ In all systems, F2B encounters high-energy barriers as it approaches the center of the membrane. Therefore, the energy in the center of the bilayer exceeds that observed in bulk water. This may indicate that the molecules spontaneously penetrate the inner membrane, anchoring beneath the lipid head groups. Interestingly, this anchoring behavior is particularly evident with F2B in sIM (Figure 3A), which reaches the global minimum at −5 kcal/mol from the water baseline. Its thermodynamic “sweet spot” is just below the head groups in the carbonyl region at 1.8 nm. Movement toward the center of the membrane is unfavorable and leads to high-energy barriers of up to 12 kcal/mol. Slightly distinct behavior was observed for F2B on the POPC membrane (Figure 3B). Like in sIM, a high barrier is noticeable once the molecule targets the center of the membrane; however, the local energy minima is flattened out. It reaches approximately −1 kcal/mol with respect to water. Thus, F2B exhibits a negligible affinity for the carbonyl component. However, it is low enough that the molecules may pass almost without restrictions between the water phase and the lipid fraction referred to. We observed a very similar behavior to the classical MD approach, where 40% of all F2B did not bind to the membrane. Hence, we can notice the selective approach of a compound to bacterial and neutral membranes. In the IM cases, we observed a firm anchoring of all molecules on the membrane surface, and the PMF study confirmed that. The “*sweet spots*” of F2B lie around the carbonyl-glycerol region just below the polar headgroups, which coincide with the density plots of F2B.

**Figure 4:**
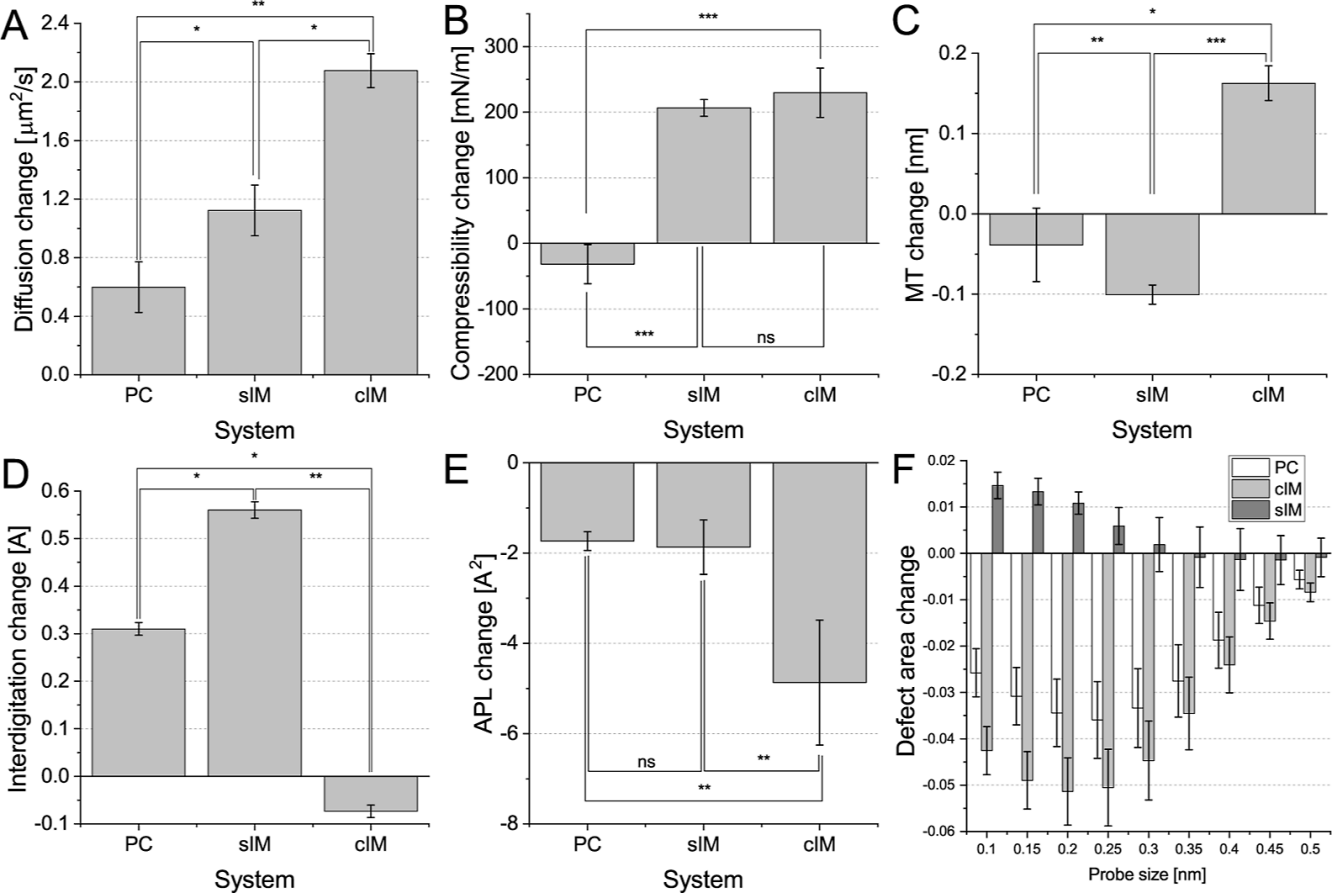
Change in membrane properties observed after the incorporation of F2B to inner membrane model systems and control POPC system. The following properties are presented (A) membrane thickness, (B) interdigitation, (C) compressibility, (D) lateral diffusion, (E) APL, and (F) defects. significance: ns—not significant, *P ≤ 0.05, **P ≤ 0.01, and ***P ≤ 0.001.

Finally, we investigated the effect of F2B incorporation on the membrane properties itself. Our study revealed a notable impact on most of the properties investigated in the system, and a few of them presented a consistent trend. Both the diffusion coefficient and compressibility exhibited a consistent trend. Regarding the diffusion coefficient, when considering a neutral PC system as a reference point, the increase in MT is more pronounced for the sIM and most prominent for the cIM system (see Figure 4A). Similarly, a decrease in compressibility was observed in the neutral PC system, while an increase was observed for both the sIM and cIM systems (Figure 4B). It should be noted that the difference between sIM and cIM is not statistically significant. Furthermore, the impact of F2B on membrane structure was assessed by examining alterations in the lipid hydrocarbon chain order parameter. The collective analysis of all *S*_cd_ profiles indicates that F2B induces a reduction in order among various lipid types within the membranes tested. Consequently, F2B introduces disruptions in the ordering of lipid tails, alters lipid diffusion, and destabilizes the pressure balance in the membrane. Thus, its effect on the membrane is similar to exposure to cationic antimicrobial peptides like magainin or anesthetic molecules such as halothane, suggesting its potential impact on lipid diffusion and lateral pressure.^58−61^ These changes may potentially result in the formation of pores and interfere with the functionality of proteins or pumps by inducing local modifications in the lipid platforms to which they are anchored.^62^

The rest of the investigated properties did not exhibit a clear and consistent trend. The incorporation of F2B in the sIM system led to a more pronounced decrease in membrane thickness compared to neutral PC. However, a contrasting trend was observed in the case of cIM (see Figure 4C). This observation aligns with the changes in interdigitation. Nonetheless, the notable disparity observed in the cIM system suggests the presence of additional factors that influence the variation in membrane thickness after exposure to F2B (Figure 4D). It is possible that F2B affected the membrane thickness, but due to the specificity of the cIM system, this was not clearly observed. The area per lipid was not significantly altered for the PC control and sIM systems, but decreased for the cIM (Figure 4E). This observed trend is unlikely to be attributed to the effect of F2B; instead, it may be linked to limitations imposed by the periodic boundary conditions (PBCs) box. Incorporation into the IM should result in an increase in the simulation box size, but in the case of the rigid OM system, such was restricted. A similar trend was also noted for defects (Figure 4F). A reduction in defect area, defined as regions that expose the acyl chain region, was observed for sIM after the incorporation of F2B in comparison to the control PC system. However, this trend was not evident for cIM, likely due to the lower density in the head region, resulting in greater exposure of the acyl chain regions, thus offsetting the effect of F2B. Alternatively, the substantial decrease in APL resulting from F2B incorporation led to the constraining of the system. Upon closer examination of Figure 4F, it becomes evident that the change in a single system significantly differs from the complex system for all probe sizes investigated. These observations underscore the need to be extremely careful when analyzing certain properties in complex systems. Detailed data on all investigated membrane properties are provided in Figures S6–S11.

### Outer Membrane

**Figure 5:**
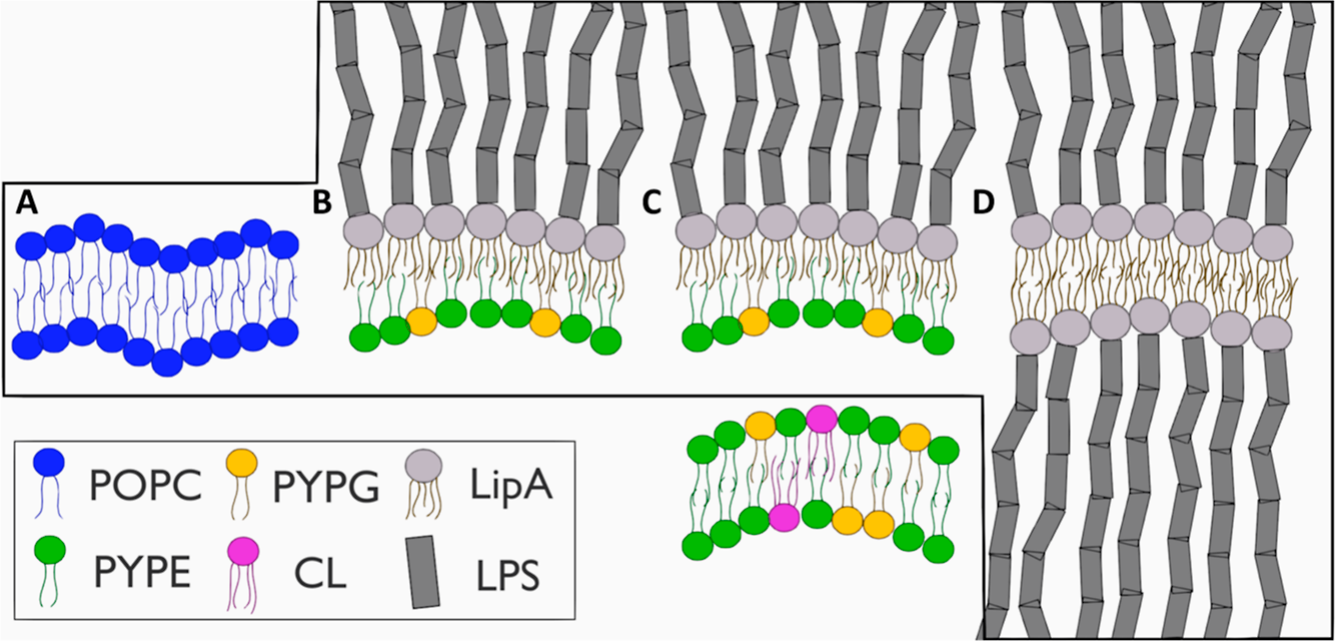
Sketch visualizing expansion of inner membrane model from (A) PC control, (B) single IM (sIM) membrane, (C) inner membrane in complex (cIM) two-membrane, and (D) LPS systems. Lipids are colored according to the type, POPC—blue, PYPE—green, PYPG—yellow, cardiolipin (CL)—violet, LipidA—gray, and LPS—dark gray. The black square represents membranes from the system analyzed in this subchapter.

Outer membrane model is less commonly used in MD studies to investigate the effect of molecules on the behavior of membranes. This could be attributed to the structural complexity of the O-antigens, which may vary among different bacterial species. Moreover, the development of accurate force field parameters for the various components of outer membranes, including O-antigens can be challenging.^63^ That proves to be demanding in simulations or the fact that the focus is especially emphasized on the behavior of membrane proteins.^64−67^ Nevertheless, it allows for assessment of the molecule’s ability to reach the membrane. As in the previous subsection, we provide a schematic representation of the analyzed systems (Figure 5) to enhance understanding and readability. In this context, a homogeneous PC system is employed as a neutral control, a single outer membrane system (sOM) represents the classical approach, and the outer membrane in a complex system (cOM) encompasses both membranes together. To provide a broader understanding of the effect of lipopolysaccharides (LPS) on the membrane, a homogeneous and symmetrical OM system (LPS) was also calculated.

**Figure 6:**
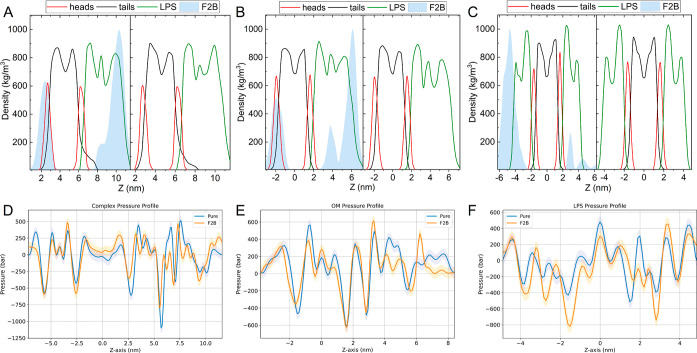
Comparison of density and lateral pressure profiles in various membrane systems. (A,D) Complex outer membrane (cOM), (B,E) single outer membrane (sOM), and (C,F) symmetric outer membrane (LPS). The upper panels illustrate the spatial distribution of molecules within each system (with and without F2B on the left and right, respectively). The F2B density was normalized in each system for clarity. The lower panels depict the corresponding pressure profiles, highlighting the variations in lateral pressure across the pure and F2B-affected membranes.

As previously mentioned, our investigation began with an analysis of the density and pressure profiles of the studied systems. In particular, there is no significant disparity in density profiles between the single outer membrane (sOM) and the combined outer membrane (cOM) systems (see Figure 6A,B). However, the distribution pattern of F2B within the outer membrane systems presents an intriguing observation. In the case of the sOM system, three distinct peaks can be discerned that represent the location of F2B (Figure 6B). These can be described in descending order of the density. The first is located in the periphery of the LPS, signifying an inability to cross the complex LPS barrier. The second peak is located in the vicinity of the PE/PG leaflet, exhibiting a pronounced affinity for the negatively charged lipid headgroups. The third peak, which is the least dense, is located at the edge of the LPS and lipid A headgroups. A similar distribution pattern is observed in the cOM system with a minor variation. In this case, the first and third peaks merge to form a slightly skewed population. Interestingly, upon examination of the symmetric LPS system, it becomes evident that the presence of F2B within the LPS is minimal. However, individual F2B particles penetrate the closest to the center of the membrane.

Similarly to previous analyses, we investigated the impact of F2B on OMs pressure profiles. The pressure profile of pure sOM aligns with findings reported in a recent article,^45^ displaying two positive peaks around the headgroup and tail region of PE–PG, reaching approximately 350 and 600 bar, respectively. Negative wells, indicative of attractive forces, are also evident in the same regions, measuring approximately −400 and 50 bar. The rest of the profile reflects the behavior at the lipidA and complex polysaccharide level. This exhibits slight variations from the literature^45^ due to the distinct structure of subsequent polysaccharide residues. Nonetheless, a noticeable trough is observed at the lipidA level, and a subsequent positive peak is observed at the beginning of the LPS structure. Notably, all OM systems experienced significant alterations in pressure distribution across the membrane (see Table 2). In the case of the cOM and sOM systems, the variations in F2B activity resulted in changes of 459 and 515 bar·nm, respectively, values comparable but slightly lower than the changes observed in a previous analysis of sIM. The symmetric membrane-LPS system was most profoundly affected, exhibiting a change equal to 827 bar·nm. This substantial difference is manifested through deeply negative wells attributed to attractive forces. Despite this, the membrane thickness remained essentially consistent. This phenomenon may be partially obscured by an increase in interdigitation or relaxation of the LPS sugar chains, especially since the positive peaks in the LPS system were only partially affected.

**Table 2:** Absolute Area under LPP for LPS, sOM, and cOM before and after F2B Exposure

		difference in PURE and F2B per leaflet [bar·nm]		
system	difference between leaflets [bar·nm]	inner leaflet	outer leaflet	pressure difference [bar·nm]
LPS				
PURE	215.22 ± 14.02	704.42 ± 37.04	122.32 ± 6.43	826.74 ± 43.47
F2B	797.32 ± 26.64			
sOM				
PURE	1817.44 ± 25.91	174.13 ± 13.11	284.82 ± 21.43	458.95 ± 34.54
F2B	1928.13 ± 28.64			
cOM				
PURE	1771.49 ± 22.91	393.08 ± 25.52	122.40 ± 7.95	515.48 ± 33.47
F2B	2286.97 ± 25.45			

In the context of our molecular dynamics simulations of OMs, we performed a thorough comparison of important membrane properties with existing literature findings. Our examination focused on key metrics such as MT, APL, and *S*_cd_ (see Figures S6–S12). The acquired data exhibited similarities with the values reported in the literature,^48,68,69^ indicating the reliability of the simulation outcomes. Our results are consistent with the APL values reported in the experimental works.^70^ Furthermore, the pressure profiles obtained closely mirrored the recent literature report.^45^ It should be noted that while the diffusion coefficients showed a small bias, this parameter is highly dependent on the chosen reference atoms in the structure, particularly regarding the length of the LPS.^5,48,71^

Subsequently, we proceeded with the characterization of the F2B behavior within the simulated membrane systems. F2B in the OM system acts slightly differently from bacterial IM models. Herein, the classical lance shape does not stand out as before; therefore, it is impossible to identify a general type of binding (see Figure S5). Exclusively for the inner leaflet (PE/PG) of the OM, we can refer to the characteristic lance shape, with the amine groups facing the water phase. When interacting with LPS, the particles individually penetrate the polysaccharide region and tend to form unstable aggregates, as well. LPS is a high-density polysaccharide barrier that can effectively limit the activity of various antimicrobial compounds. Furthermore, negatively charged residues can effectively attract positive charged compounds through electrostatic interactions.^72^ In our approach, these challenges are also evident; hence, F2B fractions are located in the R1 core. Most of the agents bonded to the d-glucose and d-galactose core; however, several individuals penetrated much deeper, reaching the d-mannoheptose region. A similar activity of F2B was observed in the sOM system. Also, the individual and aggregation scheme was identified, while the latter seems to be stable for about 100 ns. Most of the clustered particles attached to d-glucosamine and d-galactose moieties, while the individuals approached deeper to the d-mannooctulosonic acid level. The interaction with the inner leaflet of the OM composed of PE and PG was analogous to that in the cOM and IM system. In the LPS system, F2B acted in the same manner as in OM systems, although here a single particle approached the lipidA level, penetrating almost the entire R1 core. These results show that F2B is capable of penetrating through the LPS barrier, even in the limited time of MD simulation. Additionally, we can observe that the LPS system exhibits stronger curvature fluctuation than other investigated systems. Despite this, we do not notice that the effect of F2B induces a significant deformation of the membrane in either direction.

**Figure 7:**
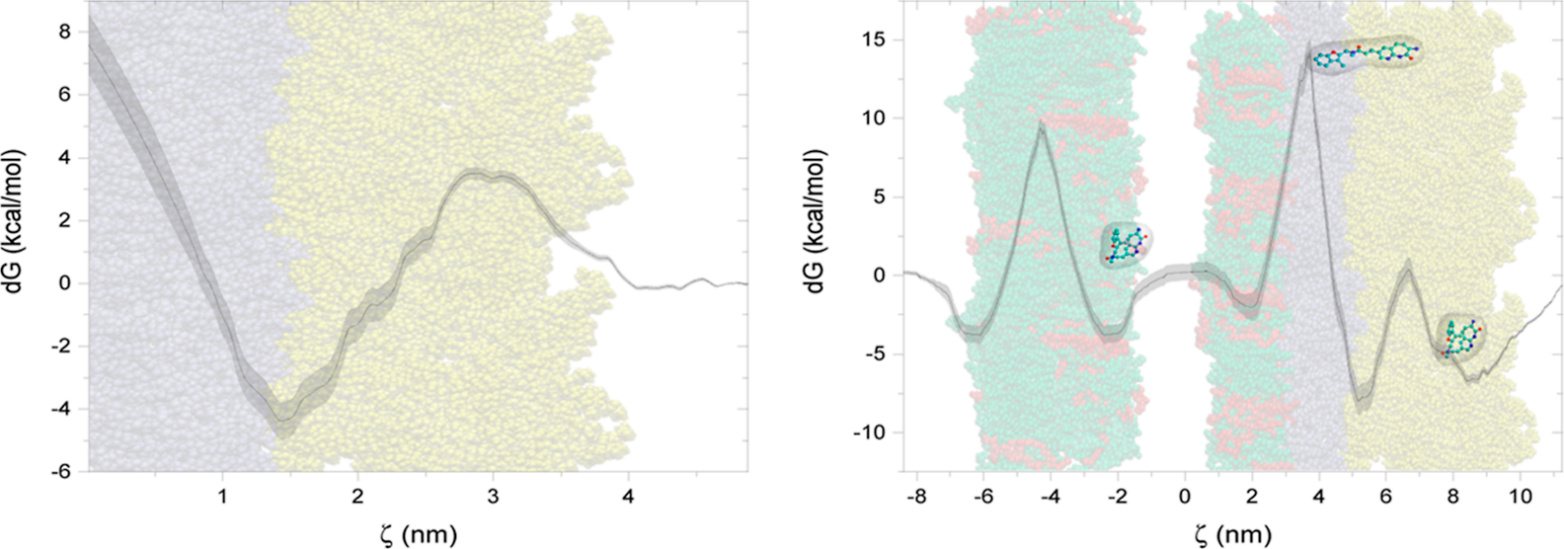
PMF profiles for F2B as a function of the distance from the center of LPS (left) and complex (right) bilayer.

As previously described, umbrella sampling analysis was performed for the investigated systems (Figure 7). As the LPS leaflet is especially interesting, we decided to focus on this aspect in our US study. The PMF obtained for the double LPS showed a different nature of the agent on the membranes compared to the IM counterpart. F2B demonstrates the ability for efficient transfer between the initial LPS region, specifically d-galactose, and water, with negligible energetic barriers. This observation aligns with our classic MD approach, where a significant presence of F2B agents was observed in this region. The first small energy barrier of 3.5 kcal/mol is faced at the d-glucose level. Subsequently, a local energy well of 8 kcal/mol is encountered in the d-mannooctulosonic surrounding in the polysaccharide core. Interestingly, few molecules were traced at that layer in our classical MD approach. Apparently, this barrier can hold a number of molecules. Nonetheless, individual molecules demonstrated the ability to anchor effectively at this level. Progressing toward the center of the OM, the particle faces a significant energy barrier, estimated to be around 12 kcal/mol from energy well. Overcoming such a barrier at the lipidA interface may pose a considerable challenge to the molecules. Thus, approaching the OM core is highly questionable.

In our examination of the cOM system, numerous parallels emerge when compared to the double LPS profile, particularly evident in the energy barriers. A distinct feature of the cOM system is the initial local minimum, presenting as −7 kcal/mol, a phenomenon absent in the LPS profile. This probably results from the incorporation of additional O antigens, which exhibit a strong affinity for binding to F2B particles. Subsequently, the cOM system encounters its first energy barrier, similar to that observed in the double LPS system. A subsequent energy well falls in the d-mannooctulosonic region, where individual molecules have also been observed in our classic MD approach. A notable peak in energy values occurs at the interface between lipid A and the outer PE–PG leaflet, where an imposing energy barrier of a total of 21 kcal/mol is observed. Subsequently, a further reduction in energy is observed, characterized by a minor energy well on the PE–PG side. When approaching the IM core, a subtle well of 4 kcal/mol becomes discernible, particularly in the phosphate layer, followed by a 12 kcal/mol increase in the acyl tail region. The behavior exhibited by F2B in both the cOM and double LPS systems reveals certain fundamental similarities regardless of the increase in free energy due to structural complexity. In the analysis of the free energy profile, a distinctive peak emerged in the central region, indicating that a substantial energy barrier encountered. This can be attributed to the charged nature of F2B, potentially leading to additional electrostatic interactions at the lipid/saccharide interface. Interestingly, analogous profiles have been documented in the literature for OM systems, suggesting that the behavior of F2B is in line with established patterns.^71,73^ The pronounced peak could also be slightly influenced by a conformational change in the molecule upon initial penetration, transitioning from a folded form within the polysaccharide region to an extended conformation at the lipidA level. Remarkably, after transversely traversing the OM, F2B reverted to its folded state, moving toward the interior of the IM.

**Figure 8:**
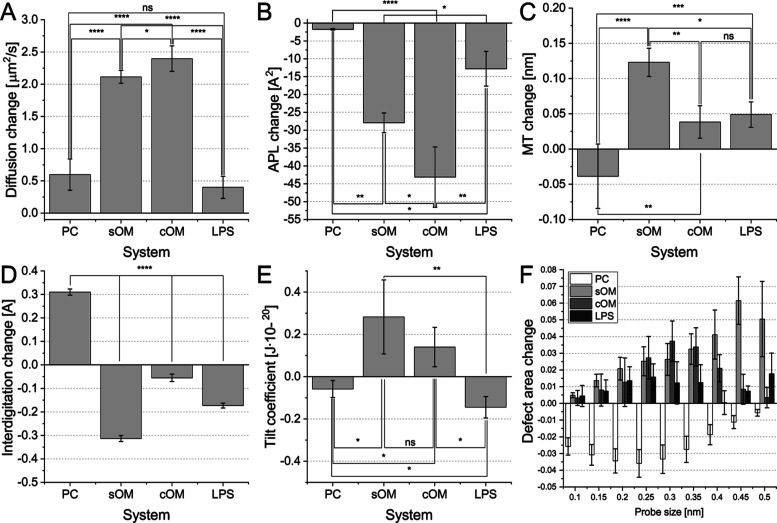
Change in membrane properties observed after incorporation of F2B to outer membrane model systems, control PC system and symmetric LPS system. The following properties are presented (A) membrane thickness, (B) interdigitation, (C) APL, (D) lateral diffusion, (E) tilt coefficient, and (F) defects. Significance: ns—not significant, *P ≤ 0.05, **P ≤ 0.01, ***P ≤ 0.001, and ****P ≤ 0.0001.

In conclusion, our investigation evaluated the influence of F2B on membrane properties, revealing consistent patterns in lateral diffusion and APL. Notably, the diffusion coefficient increased consistently following the incorporation of F2B into the membrane, as shown in Figure 8A, mirroring observations in the context of IM. Furthermore, a consistent decrease in APL was observed, as demonstrated in Figure 8B. While some differences were observed between the PC and OM systems in certain properties, the trend varied between the sOM and cOM systems. This is evident in the case of MT, where an increase was observed in OM systems after incorporation of F2B with a higher increase observed in the sOM system (see Figure 8C). Interestingly, interdigitation exhibited a reverse trend (Figure 8D), indicating that changes in OM systems are more closely related to system behavior than the increased interleaflet interaction. Nevertheless, considering the magnitude of the differences in interdigitation and membrane thickness between the sOM and cOM systems, it can be concluded that interleaflet interactions play a role in these alterations to some extent.^74^ Notably, changes in membrane thickness correlated with alterations in tilt (Figure 8E). In contrast, no discernible trend was observed in compressibility of the OM systems, unlike the IM systems.

We also performed a defect analysis in the OM model systems (Figure 8F). Similarly to IM findings, there were differences in defect characteristics between single and complex systems. In the case of the sOM system, the incorporation of F2B resulted in an increased occurrence of defects. This is not surprising, considering that all F2B molecules were incorporated into the PE/PG leaflet in the acyl/head region. Consequently, a subsequent relaxation of lipids was expected in the opposite leaflet, increasing the level of exposition of the acyl region (defects). The trend observed in the cOM system was consistent, although to a different extent. In particular, for probe sizes ranging between 0.25 and 0.35, the effect in the cOM system was slightly stronger than that even in the sOM system. Furthermore, a notable increase in the presence of defects was observed for probe sizes ranging from 0.1 to 0.35 in the symmetrical LPS system. This finding is intriguing considering that only a single F2B molecule approached the lipid A region. It suggests that disruptions within the LPS region do indeed influence membrane behavior.^75^

## Discussion and Conclusions

There are multiple well-established mechanisms that facilitate antimicrobial effects. These mechanisms range from inhibiting specific chemical or biological pathways, to obstructing ion pumps, and even altering membrane structures to a degree that compromises their functional integrity.^76,77^ The structure of F2B can also play a pivotal role in its antibacterial activity. Considering the conclusions from the structure–activity relationship (SAR) work, it can be observed that the presence of a positively charged amino group may facilitate electrostatic interactions with negatively charged components on the surface of the bacterial cell. This interaction has the potential to disrupt membrane integrity or interfere with essential cellular processes.^78−80^ Furthermore, cyclic aromatic rings in the F2B structure can allow for π–π interactions, enhancing its ability to bind to specific molecular targets within bacterial cells.^81,82^ Moreover, the balanced distribution of the hydrophobic and hydrophilic regions may play an important role in its activity. Our simulations revealed a consistent orientation of agent in planar membranes, with benzofuran rings facing the bilayer center and amine groups toward the polar lipid headgroups.

The impact of the novel antimicrobial molecule F2B on cellular membranes has not yet been addressed. This study investigates whether the observed antimicrobial efficacy of F2B is attributed in part or exclusively to the disruption of membrane homeostasis, defined as the maintenance of specific membrane properties.

To this end, we formulate a series of hypotheses that aim to elucidate the interaction level between F2B and membrane structures. Our primary hypothesis posited that F2B would exhibit more pronounced interactions with bacterial membrane systems (IM or OM) compared with the control PC system. Our findings, as indicated by the PMF analysis, support this hypothesis. The PMF exhibited a local minimum of approximately −1 kcal/mol for the PC system (see Figure 3), in stark contrast to the −4.5 and −8 kcal/mol observed for the inner and outer bacterial membranes, respectively (see Figures 3 and 7). This differential interaction is further evident in the behavior of the molecules within these systems. In bacterial systems, F2B molecules penetrate more deeply, while in a neutral system they tend to cluster in a water environment, exhibiting limited integration with the membrane.

Upon confirmation that F2B demonstrates enhanced interactions with bacterial systems, our research progressed to the evaluation of the second hypothesis that F2B significantly impacts at least one aspect of what we call membrane homeostasis. Our findings reveal notable alterations in the properties of the IM system after incorporation of F2B, specifically in aspects such as compressibility, lateral diffusion, and leaflet pressure. Similarly, the OM exhibited substantial changes in various properties, including the area per lipid, tilt coefficient, and lateral diffusion. These results confirm the hypothesis that F2B significantly influences local membrane homeostasis in both the IM and OM models. Notably, phenomena such as alterations in membrane thickness,^83^ modifications in protein lateral diffusion,^84^ and changes in tilt and compressibility, which are correlated with membrane order and resilience,^59^ together with the antimicrobial effect were reported.

Subsequently, we focused more on complex systems. We hypothesized that these systems would more accurately reflect molecular behavior and that their close presence in bacteria must involve some degree of coinfluence. Although this approach is infrequently utilized, the findings indicate that such constructed systems exhibit variations in diffusion and induce lipid sorting, through the presence of proteins.^85^ Furthermore, in stratum corneum lipid membranes, it was observed that such complex systems can spontaneously self-assemble.^86^ In our case, in the comparison of pure systems (without F2B), notable differences were observed between complex and single ones. Specifically, the density in the headgroup region of the complex system was lower compared with the corresponding single system. This was true for both OM and IM. In the cIM model, an increased density in the acyl chain region was also observed. Additionally, the interleaflet pressure in both complex systems was lower than that for the corresponding single ones. We also identified several disparities in membrane properties, including apparent bending rigidity, which was significantly different in complex systems compared with single systems. For IM, this rigidity was notably higher, whereas for OM slightly decreased (see Figure S9). Lateral diffusion was also significantly higher in complex systems (Figure S7). Furthermore, differences in membrane thickness, compressibility, and tilt energy were observed, although only in the OM system.

After the incorporation of F2B, we observed some differences between single and complex systems for both IM and OM layers. In OM systems, changes in MT, APL, interdigitation, and diffusion were more pronounced in the complex system than in the single one. Similarly, in IM systems, the changes were more significant in cIM than in sIM for APL, diffusion, and compressibility. However, the trend for MT and defects was inverse in cIM compared to that in sIM. The opposite situation was noted for OM systems. While we speculate that differences in APL and defects may be attributed to technical limitations (as PBC cannot be independently adjusted to different membranes within the system), the observed variations in other properties remain significant.

Finally, we examined the lipopolysaccharide (LPS) barrier, which is a critical challenge for any antimicrobial molecule. Contrary to our initial hypothesis, significant differences were also observed in symmetric LPS systems compared to those of the corresponding sOM and cOM systems. These discrepancies might partially arise from the lack of asymmetry in the LPS layers as well as the shorter O-antigen region. After F2B incorporation, we observed different impacts on membrane properties: reduced changes in lateral diffusion, reversed alterations in tilt energy, and limited change in MT similar to those of other OM systems. The latter may arise due to the hindered incorporation of F2B molecules into the lipid phase by the LPS region. Notably, in the complex system, PMF analysis revealed a commentable energy barrier at the LPS layer, indicating some limitations for the investigated molecule to reach the lipid core. Regarding simulation-specific methodologies, employing a double-bilayer configuration presents challenges to the penetration process. This is because the lateral dimensions of the two membranes are coupled through the simulation box and the PBC. Consequently, the agent translocation is not tensionless, as ideally required. The tension generated by the double bilayer setup increases the free energy barrier for translocation. Possibly, larger systems would allow one to minimize this bias.^87^ Despite that in classical MD, F2B demonstrated remarkable penetration capabilities to pass through the LPS region, as evidenced in density plots. Consequently, our study reveals distinct differences in membrane properties between the modeled systems, highlighting F2B ability to penetrate the OM layers but limited efficacy in passing through the lipidA membrane region within the observed simulation duration.

In conclusion, the impact of F2B on the homeostasis of membrane properties indicates that its activity extends beyond the conventional targeting of the FabI enzyme and highlights a multifaceted potential as a versatile agent to combat bacterial infections. The systematic changes observed in lipid membranes due to F2B incorporation reveal a substantial impact on the membrane functionality. The role of membranes in F2B activity has been neglected so far, but our studies may suggest that they may also be significant in their overall antimicrobial effect.

## Data Availability

MD simulations and trajectory analyses were performed using commonly used programs, i.e., GROMACS or VMD. Analyses were supported and performed with Python and Matlab scripting. All procedures employed in this work are described in the Methods section. All system information, input, and parameter files are available in the Zenodo repository (10.5281/zenodo.10946177) and the Supporting Information.

## Supporting Information Available

Membrane system characteristics; individual properties determined for membrane systems such as membrane thickness, area per lipid, interdigitation, lateral diffusion coefficient, compressibility, tilt bending coefficient, apparent bending rigidity, lipid tails order parameter, and membrane curvature; F2B orientations in various membrane systems; and umbrella sampling histograms (PDF)
F2B topology files (ZIP)
