# Supplementary material for: Multifaceted Activity of Fabimycin: Insights from Molecular Dynamics Studies on Bacterial Membrane Models
Source: J Chem Inf Model. 2024 May 11;64(10):4204–17. doi: 10.1021/acs.jcim.4c00228 (PMC11134499; doi:10.1021/acs.jcim.4c00228)
Supplement: Supplementary file 1 — ci4c00228_si_001.pdf [file ci4c00228_si_001.pdf]

Supporting Information: Multifaceted Activity of Fabimycin: Insights from Molecular Dynamics Studies on Bacterial Membrane Models

Mateusz Rzycki* and Dominik Drabik

Department of Biomedical Engineering, Wroclaw University of Science and Technology, Wroclaw, 50-370, Poland

E-mail: mateusz.rzycki@pwr.edu.pl

Systems Characteristics

In our study, we constructed various lipid membrane systems with CHARMM GUI,[1–4] each tailored for specific membrane reflection. The systems included a phosphatidylcholine (PC) bilayer, denoted as a control system and bacterial mimicking such as single inner membrane (sIM), asymmetric single outer membrane (sOM), symmetric outer membrane (LPS) and a complex hybrid dual membrane system with inner and outer membrane included (complex). The sIM system was created using a combination of phosphatidylethanolamine (PYPE 16:0 / 16:1), phosphatidylglycerol (PYPG 16:0 / 16:1), and cardiolipin (PVCL2 16:0,16:0 / 18:1,18:1) lipids.[5] sOM was made up of PYPE, PYPG and the *E. coli* lipopolysaccharide (LPS) composed with LipidA type1, R1 core, and 2 repeating units of the O6-antigen.[6] The length of the O6-repeating units was adapted based on the results published by Wu *et. al*[7,8] and Rzycki *et. al*.[6] The number of LPS molecules and phospholipids was adjusted equally to the total lipid area occupied in each leaflet. Ca^{2+} ions were automatically added based on the length of the LPS to neutralize the system. LPS system was created with equally distributed *E. coli* LPS molecules composed of LipidA type1 and R1 core.

Table S1: Characteristics of simulated membrane systems

**System type**	**Number and type of lipids**	**Avg. simulation time per replica** [μs]
**PC**	400 POPC, 24443 TIP3	0.4
**POPC F2B**	400 POPC, 24 F2B, 23614 TIP3	0.6
**sIM**	160 PYPE, 30 PYPG, 10 PVCL, 20000 TIP3	0.7
**sIM F2B**	160 PYPE, 30 PYPG, 10 PVCL, 24 F2B, 19190 TIP3	0.6
**sOM**	26 LPS, 61 PYPE, 21 PYPG, 15401 TIP3	0.4
**sOM F2B**	26 LPS, 61 PYPE, 21 PYPG, 24 F2B, 15002 TIP3	0.8
**LPS**	60 LPS, 9079 TIP3	0.4
**LPS F2B**	60 LPS, 24 F2B, 13584 TIP3	0.8
**complex**	26 LPS, 185 PYPE, 45 PYPG, 8 PVCL, 16155 TIP3	0.4
**complex F2B**	26 LPS, 185 PYPE, 45 PYPG, 8 PVCL, 36 F2B, 15333 TIP3	0.8

The dual complex system was constructed from the sOM and sIM systems separated by approx. 4nm water slab mimicking the periplasm. Some of the systems were enlarged (>200 lipids) to verify whether membrane parameters may depend on the size of the system and the concentration of fabimycin (F2B) (see Figure S1). In all listed systems, F2B molecules were placed in the water phase 15-20 Å from the surface of the bilayer. The detailed specifications of the bilayer systems along with their production time are presented in Table S1.

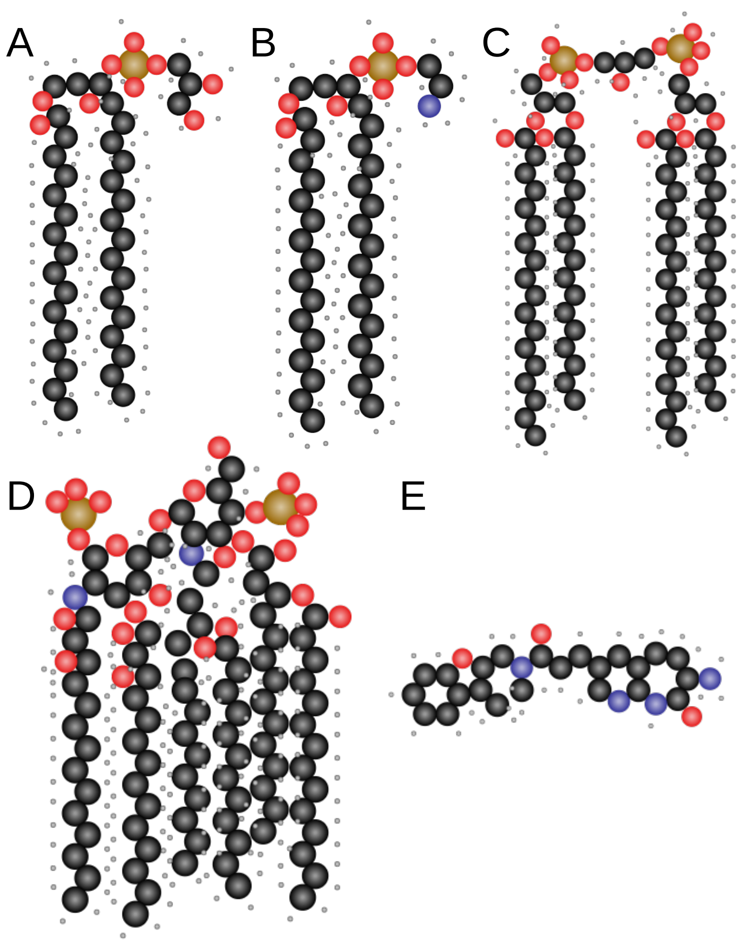

Figure S1: Structural representation of lipids and molecules employed in this study; A) PYPG, B) PYPE, C) PVCL, D) LipidA, E) F2B. Carbon, oxygen, phosphorous, and hydrogen atoms are colored in black, red, yellow, and grey, respectively.

System Equilibration

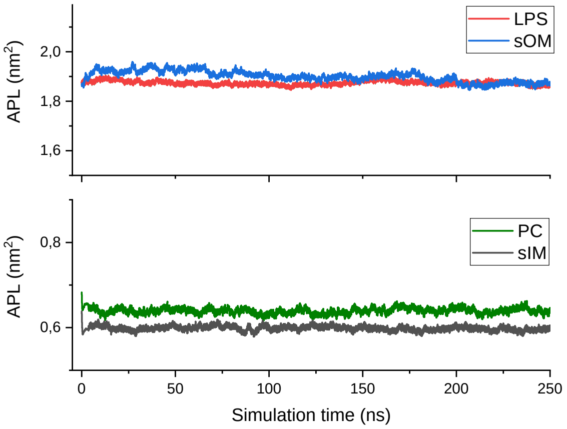

Figure S2: Surface area per lipid as a function of time for the IM, PC, OM, LPS systems.

Umbrella histograms

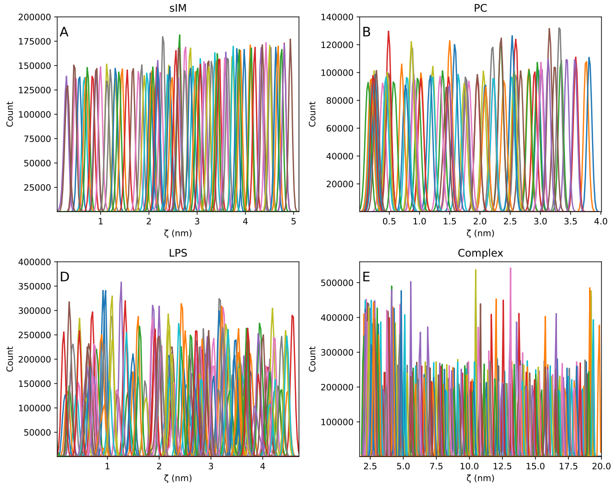

Figure S3: Umbrella sampling histograms generated during the calculation of the potential mean force. The aim is to determine the free translocation energies associated with the permeation of fabimycin across the (A) inner bacterial membrane (IM), (B) the PC membrane, (C) the symmetrical outer bacterial membrane (LPS) and (D) the double complex system (complex).

F2B orientation

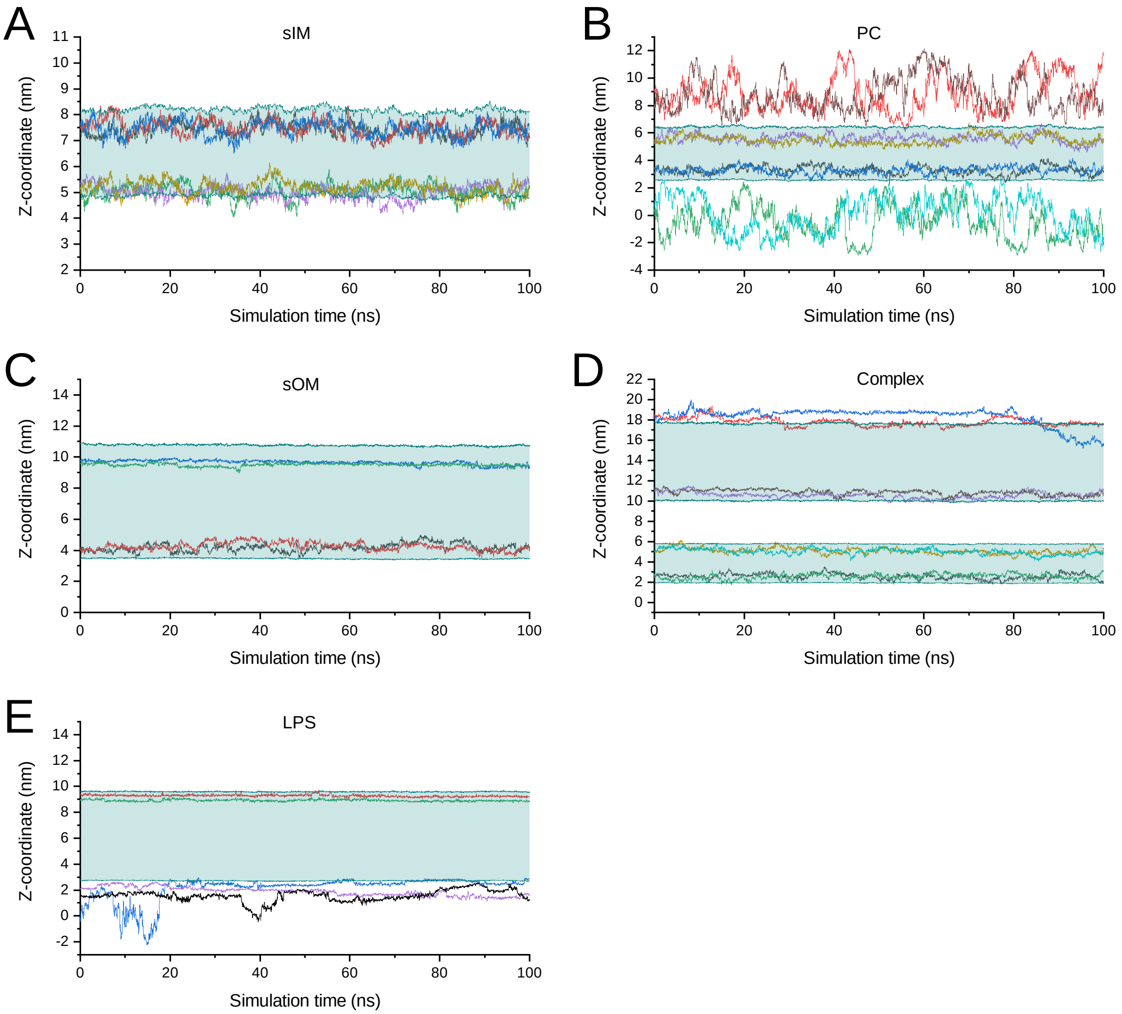

Figure S4: Location of several F2B molecules in the last 100ns of the simulation. The membrane core including the phosphorus atoms have been marked in light blue, while the remaining F2B molecules are marked in the corresponding other colors A) sIM, B) PC, C) sOM, D) Complex, E) LPS systems.

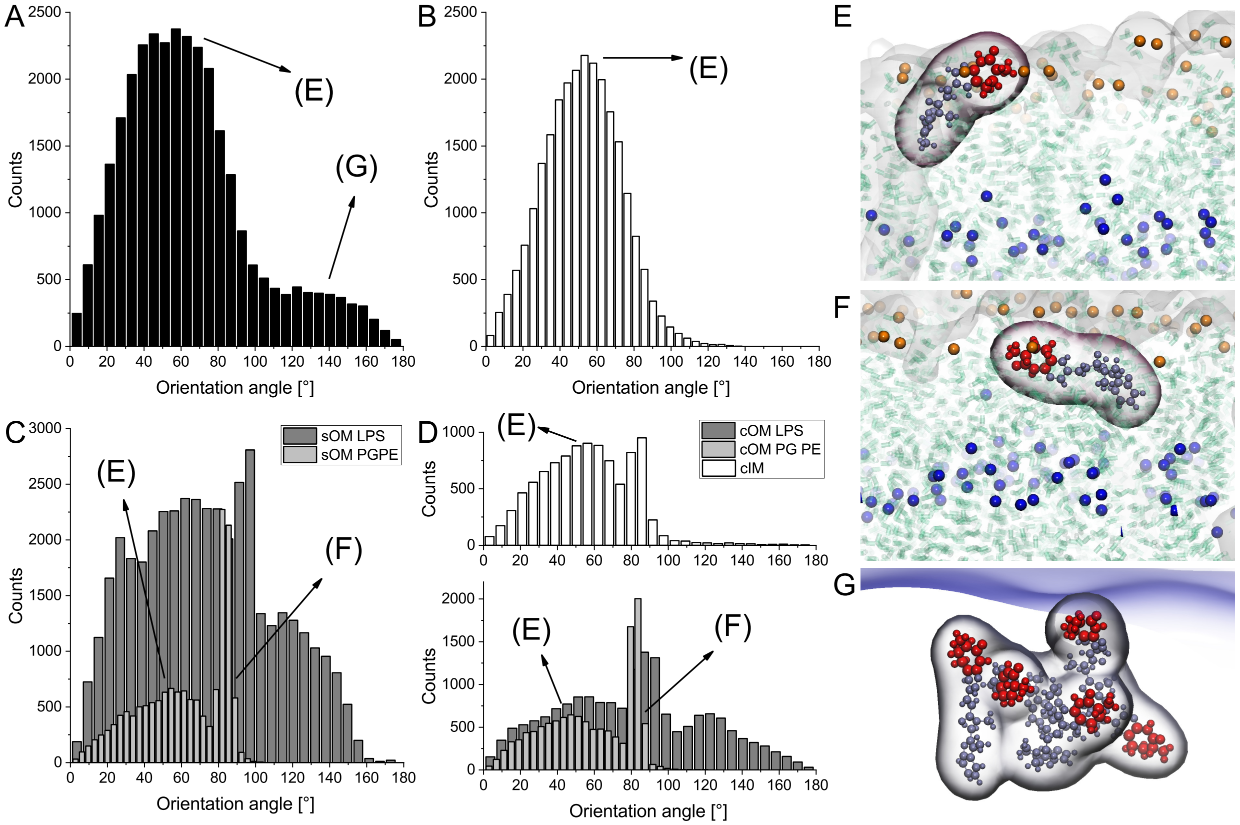

Figure S5: Distributions of angles between F2B molecule (defined as a vector between N4 and C10 atoms) and normal to the leaflet for (A) POPC, (B) sIM, (C) sOM and (D) complex systems. In panels (E-G), snapshots of the most frequent conformations of F2B are presented. In the snapshots, the charged amine fragment of F2B is highlighted in red. Orange beads indicate the position of phosphorus, while blue beads represent the last carbon atoms in the acyl chain (C16). Due to the dense structure of the LPS in sOM and cOM leaflets, the limited interaction of F2B with lipidA occurs, thus the distribution angle covers the entire orientation range.

Individual parameters of membrane systems

In main section the parameters are represented as change observed due to incorporation of F2B. In this section absolute values of determined parameters are presented. Specifically, membrane thickness and area per lipid are presented in Figure S6; interdigitation and lateral diffusion coefficient are presented in Figure S7; area compressibility and tilt coefficient are presented in Figure S8, membrane curvature are presented in Figure S11; acyl chain order parameter are presented in Figure S10 and S12. Finally, both apparent bending rigidity and bending rigidity are presented in Figure S9. This distinction is caused by the fact that bending rigidity determined using real-space fluctuation method for smaller systems (≤ 200 lipids) returns underestimated values. It is still possible to investigate the change on this parameter, however the absolute values are not related to the experimental ones. To this end term 'bending rigidty' was only used in the case of larger systems.

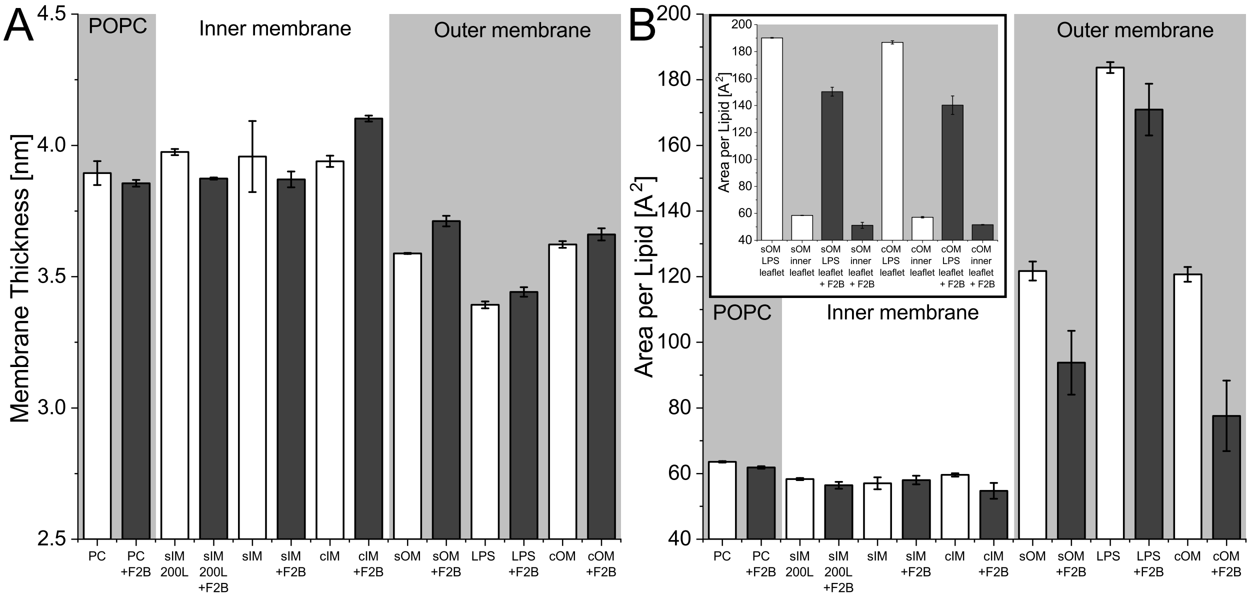

Figure S6: Absolute values of (A) membrane thickness and (B) area per lipid of investigated membrane systems. Since values for outer membranes are presented as average of both leaflets, an inset is added. In the inset distinction between the leaflets is presented.

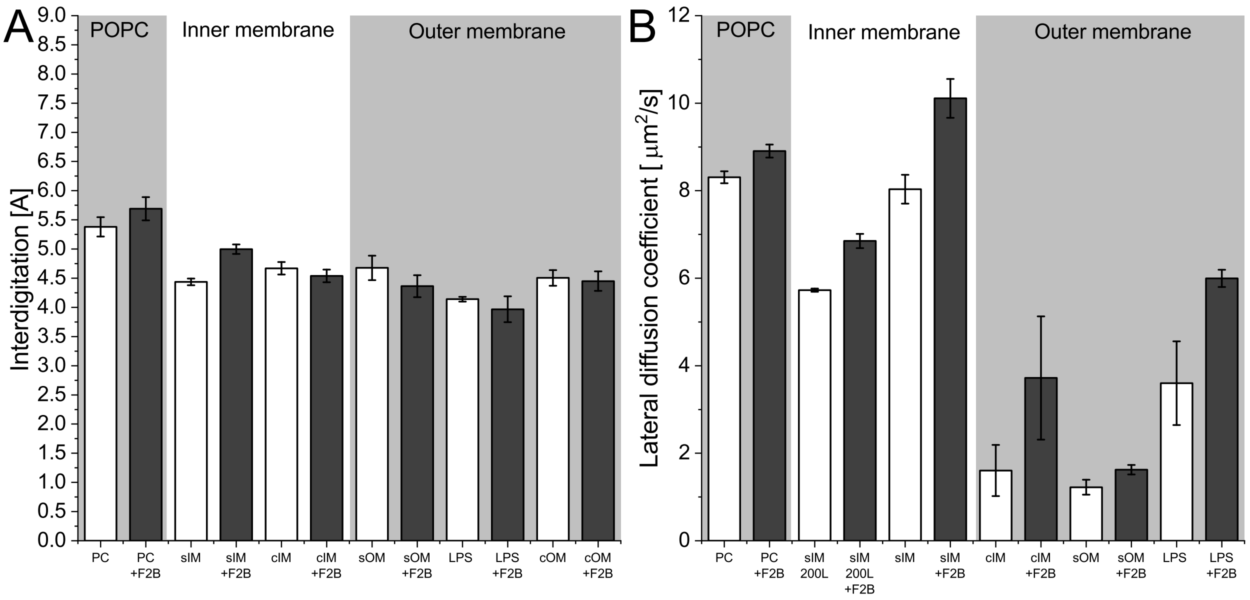

Figure S7: Absolute values of (A) interdigitation and (B) lateral diffusion coefficient of investigated membrane systems.

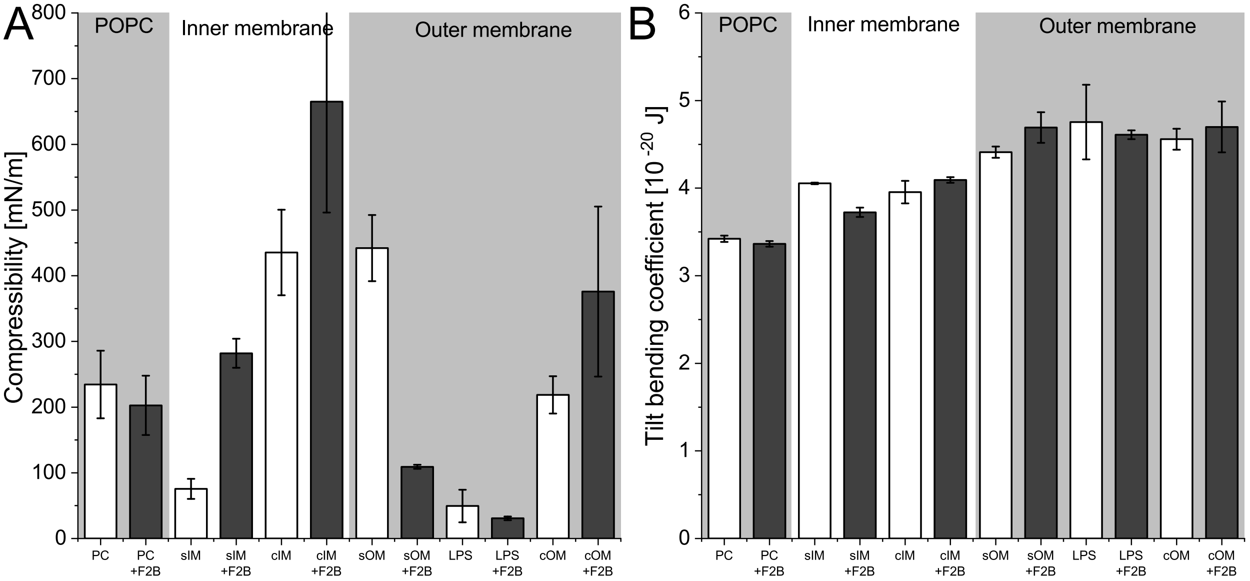

Figure S8: Absolute values of (A) compressibility and (B) tilt bending coefficient of investigated membrane systems.

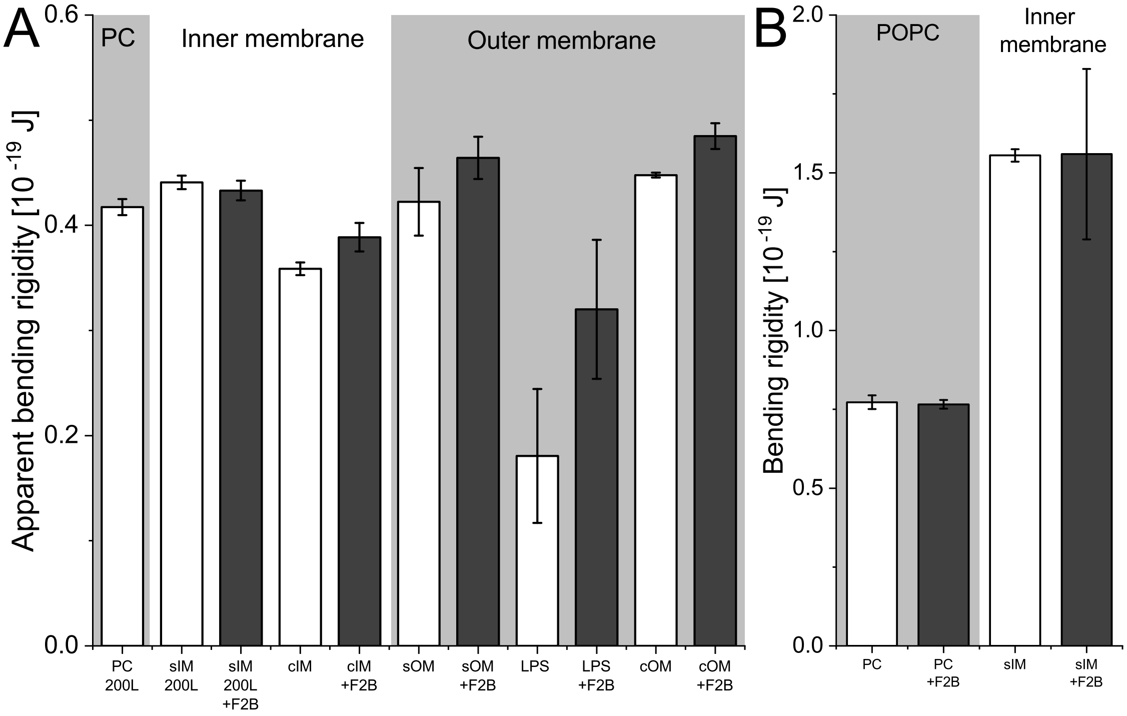

Figure S9: Absolute values of (A) apparent bending rigidity and (B) real bending coefficient calculated for larger system of investigated membrane systems.

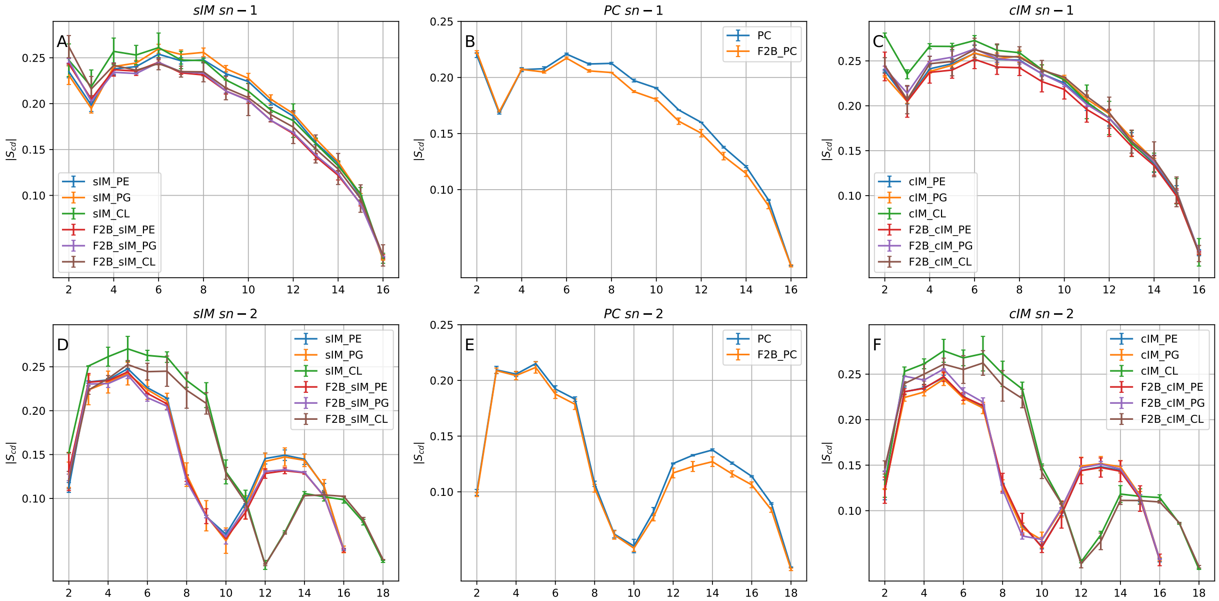

Figure S10: Order parameters $|S_{cd}|$ for saturated $sn-1$ (top) and unsaturated $sn-2$ (bottom) acyl chains of sIM (A,D panels), PC (B,E panels), cIM (C,F panels) systems. Low carbon atom numbers correspond to those close to the headgroup.

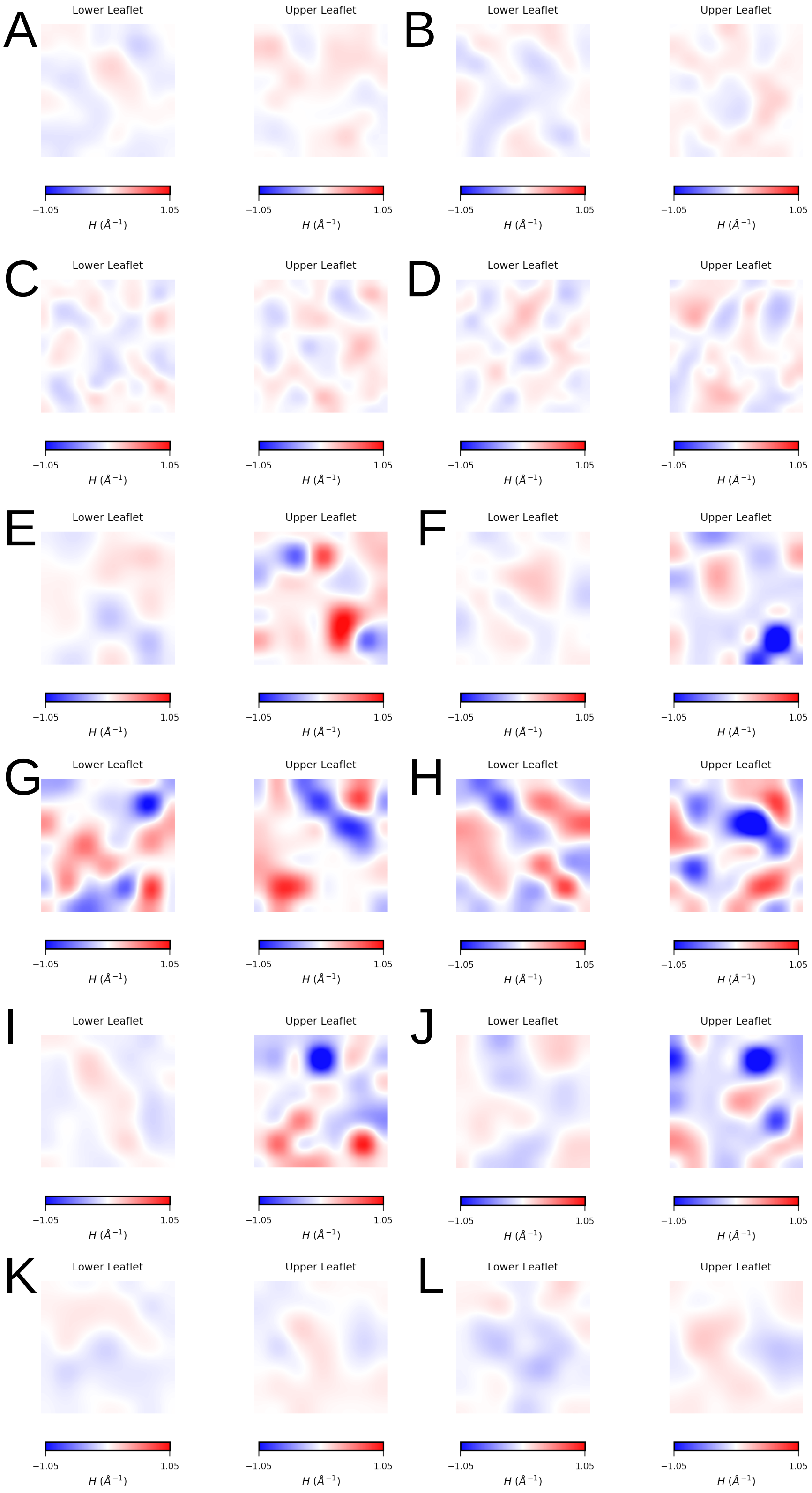

Figure S11: Mean curvature H of all leaflets in tested membranes; A) IM B) IM F2B, C) PC, D) PC F2B, E) OM, F) OM F2B, G) LPS, H) LPS F2B, I) Complex (OM), J) Complex F2B (OM), K) Complex (IM), L) Complex F2B (IM).

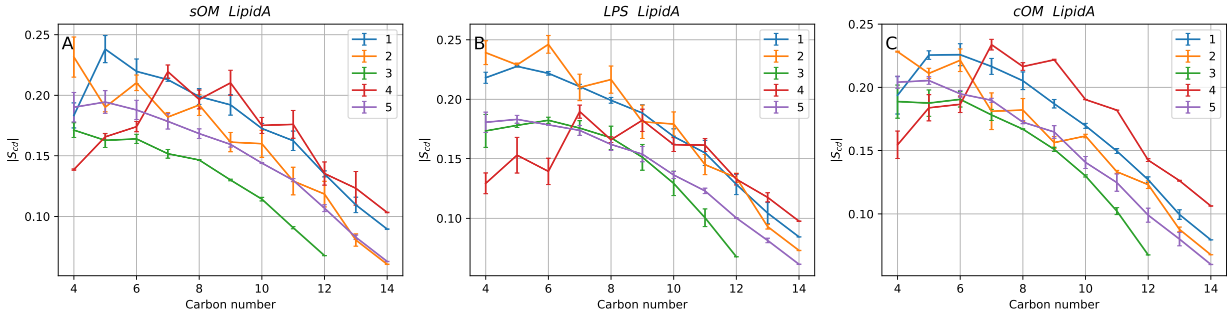

Figure S12: Order parameters $|S_{cd}|$ for subsequent lipidA acyl chains of sOM (A), LPS (B), cOM (C) systems. Low carbon atom numbers correspond to those close to the headgroup.

References

(1) Feng, S.; Park, S.; Choi, Y. K.; Im, W. CHARMM-GUI Membrane Builder: Past, Current, and Future Developments and Applications. *J. Chem. Theory Comput.* **2023**, *19*, 2161–2185.

(2) Lee, J. et al. CHARMM-GUI Membrane Builder for Complex Biological Membrane Simulations with Glycolipids and Lipoglycans. *J. Chem. Theory Comput.* **2019**, *15*, 775–786.

(3) Jo, S.; Lim, J. B.; Klauda, J. B.; Im, W. CHARMM-GUI Membrane Builder for Mixed Bilayers and Its Application to Yeast Membranes. *Biophys. J.* **2009**, *97*, 50–58.

(4) Lee, J. et al. CHARMM-GUI Input Generator for NAMD, GROMACS, AMBER, OpenMM, and CHARMM/OpenMM Simulations Using the CHARMM36 Additive Force Field. *J. Chem. Theory Comput.* **2016**, *12*, 405–413, PMID: 26631602.

(5) Rzycki, M.; Drabik, D.; Szostak-Paluch, K.; Hanus-Lorenz, B.; Kraszewski, S. Unraveling the mechanism of octenidine and chlorhexidine on membranes: Does electrostatics matter? *Biophys. J.* **2021**, *120*, 3392–3408.

(6) Rzycki, M.; Kraszewski, S.; Drabik, D. Towards Mimetic Membrane Systems in Molecular Dynamics: Characteristics of E. Coli Membrane System. *Lect. Notes Comput. Sci.* **2021**, *12743 LNCS*, 551–563.

(7) Wu, E. L.; Engström, O.; Jo, S.; Stuhlsatz, D.; Yeom, M. S.; Klauda, J. B.; Widmalm, G.; Im, W. Molecular Dynamics and NMR Spectroscopy Studies of E. coli Lipopolysaccharide Structure and Dynamics. *Biophys. J.* **2013**, *105*, 1444–1455.

(8) Wu, E. L.; Fleming, P. J.; Yeom, M. S.; Widmalm, G.; Klauda, J. B.; Fleming, K. G.; Im, W. E. coli outer membrane and interactions with OmpLA. *Biophys. J.* **2014**, *106*, 2493–2502.
